# Development of Non-Natural Flavanones as Antimicrobial Agents

**DOI:** 10.1371/journal.pone.0025681

**Published:** 2011-10-19

**Authors:** Zachary L. Fowler, Karan Shah, John C. Panepinto, Amy Jacobs, Mattheos A. G. Koffas

**Affiliations:** 1 Praxair, Inc. BioPharma Research and Development, Burr Ridge, Illinois, United States of America; 2 Department of Chemical and Biological Engineering, University at Buffalo, the State University of New York, Buffalo, New York, United States of America; 3 Department of Microbiology and Immunology, University at Buffalo, the State University of New York, Buffalo, New York, United States of America; 4 Department of Chemical Engineering, Rensalear Polytechnic Institute, Troy, New York, United States of America; National Institutes of Health, United States of America

## Abstract

With growing concerns over multidrug resistance microorganisms, particularly strains of bacteria and fungi, evolving to become resistant to the antimicrobial agents used against them, the identification of new molecular targets becomes paramount for novel treatment options. Recently, the use of new treatments containing multiple active ingredients has been shown to increase the effectiveness of existing molecules for some infections, often with these added compounds enabling the transport of a toxic molecule into the infecting species. Flavonoids are among the most abundant plant secondary metabolites and have been shown to have natural abilities as microbial deterrents and anti-infection agents in plants. Combining these ideas we first sought to investigate the potency of natural flavonoids in the presence of efflux pump inhibitors to limit *Escherichia coli* growth. Then we used the natural flavonoid scaffold to synthesize non-natural flavanone molecules and further evaluate their antimicrobial efficacy on *Escherichia coli*, *Bacillus subtilis* and the fungal pathogens *Cryptococcus neoformans* and *Aspergillus fumigatus*. Of those screened, we identified the synthetic molecule 4-chloro-flavanone as the most potent antimicrobial compound with a MIC value of 70 µg/mL in *E. coli* when combined with the inhibitor Phe-Arg-ß-naphthylamide, and MICs of 30 µg/mL in *S. cerevesiae* and 30 µg/mL in *C. neoformans* when used alone. Through this study we have demonstrated that combinatorial synthesis of non-natural flavonones can identify novel antimicrobial agents with activity against bacteria and fungi but with minimal toxicity to human cells.

## Introduction

Bacterial pathogens often develop mechanisms of resistance to different chemical compounds over time by adapting their transporter systems to remove toxic compounds, such as drugs and detergents, a mechanism identified over thirty years ago while screening cancer drugs [1], [2]. It has been established that efflux pumps, particularly the resistance–nodulation–cell division (RND) type CmeABC pump, have an important role in the antimicrobial resistance of a number of pathogenic species [3], [4]. Pathogenic strains, of species such as *Pseudomonas aeruginosa*, *Escherichia coli*, *Staphylococcus aureus*, and *Salmonella enterica*, that develop broad substrate profiles become what is known as multidrug-resistant (MDR) organisms and are a particular hazard to modern medical practices. The latest antibacterial treatments are being developed by screening collections of compounds against a panel of bacterial pathogens in their MDR form and RND mutants to identify improved or novel antibacterial activity [5].

Recent habits in the American diet has shown increased interest in adopting healthy eating behaviors based on Asian and Mediterranean diets in an effort to reduce their risk of heart disease, diabetes, cancer and other lifestyle related disorders [6]. These same diets are often rich in plant polyphenols, such as flavonoids and carotenoids, due to the increased consumption of fruits and vegetables often involved in such diets. Flavonoids in particular are known to have diverse pharmico-dynamic abilities, which has drawn major attention to the molecules for both personal health applications [7], [8], and from pharmaceutical companies keen on either their native nutraceutical properties [9]. Increases to levels of polyphenolic substances have been demonstrated in plants during fungal infection, suggesting that these molecules may be part of the host-defense mechanism [10], [11].

Several recent reviews and studies have reported the antimicrobial efficacy of these molecules. Specifically, chalcones hybrids have been shown to be potent against a range of organisms including *Staphylococcus aureus* [12], [13]. Numerous other studies have identified antimicrobial activities of other flavonoid classes as well [11], [14], [15], [16], [17], [18], [19]. Despite these efforts, flavonoids and flavonoid-like molecules have failed to become treatment options due to several factors including combinatorial-potency, molecule toxicity and the fact that sufficiently large polyphenol libraries are hampered by a low abundance in plant material or difficult purification strategies. However, recent work has demonstrated that plant natural products can be produced in large and highly pure quantities using recombinant production platforms [20], [21], [22].

Since the antimicrobial effectiveness is thought to come from flavonoids abilities to form complexes with both extracellular and soluble proteins as well as bacterial membranes [23], [24], penetration into a cell and the maintenance of intracellular concentrations in infecting species becomes a critical concern for the development of flavonoids as the next generation of antibacterial/fungal agents. In light of this we first aimed to identify the natural potency of flavanones to limit *E. coli* growth in the absence and presence of two RND efflux pump inhibitors. Then, after creating a set of B-ring substituted, non-natural flavanones with different functional groups, they were similarly screened for their potency to stop or impede growth of not only *E. coli*, but a range of organisms that included *Bacillus subtilis*, *Saccharomyces cerevisiae*, and the fungal pathogens *Cryptococcus neoformans*, *Aspergillus fumigatus*.

## Materials and Methods

### Chemical synthesis of non-natural flavanones

**Figure 1:**
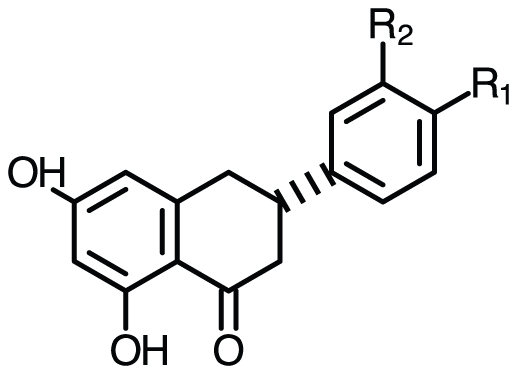
Flavanone chemical structure. Non-natural flavanone structures have functional groups attached to the B-ring in either the 4- (R_1_) or 3- (R_2_) position.

Benzaldehyde starting molecules were purchased from Sigma Aldrich and Alfa Aesar. silica gel and sand were ordered from Sigma Aldrich while the organic solvents were purchased from EMD Biosciences. Chemical synthesis of synthetic flavanones has been previously performed [25], [26] and recently reviewed [27]. Briefly, the first steps include the production of a purified and protected acetophenone from the 2,4,6-trihydroxy acetophenone monohydrate. Following de-protection, acetophenone is combined through a Claisen-Schmidt condensation with a slight access of a benzaldehyde containing a substitution of interest to be displayed in the B-ring. This results in the formation of the pre-flavonoid intermediate chalcone, which is then purified and de-protected overnight in HCl and methanol. A pH adjustment is then used to carryout the ring closure to synthesize the final flavanone molecule with the B-ring substitution. Solvent evaporation is then used to obtain crystals of desired flavanone molecule to be purified by flash chromatography. Verification of flavonoids was done using HPLC having retention times between 12 and 17 minutes using a solvent system and C18 column previously described [28]. The flavanone structure is shown in Figure 1 with functional groups attached to the B-ring in either the 4- (R_1_) or 3- (R_2_) position.

### Strains and culture media

Bacterial strains *Eschericha coli* BW25113 and *Bacillus subtilis* JH642 and the yeast *Saccharomyces cerevisiae* INVSc1 were obtained from laboratory stocks. Clinical isolates of *Cryptococcus neoformans* H99 and *Aspergillus fumigatus* H237 were used from prior laboratory isolations. HeLa cells were purchased from ATCC as a model human cell line for toxicity testing.

Luria Broth (LB) from Sigma Aldrich was used for the growth of *E. coli* strain while a homemade LB containing 10 g/L peptone, 10 g/L NaCL, and 5 g/L yeast extract (Difco) was used to culture *B. subtilis*. YPD media (Difco) was used for cultures of *S. cerevisiae*. Culturing of *C. neoformans* and *A. fumigatus* was done in a filter sterilized synthetic dextrose (SD) media containing 0.57% yeast base nutrients without amino acids (Difco) and 2% dextrose (Fisher). Dulbeco's Modified Eagle Medium (DMEM) media containing 10% FBS and 1% glutamine was used for HeLa cell cultures.

### Potency assay and CFU assay

The cell densities of overnight cell cultures for bacterial or fungal strains were first measured and then diluted to reach a normalized optical density at 600 nm (OD_600_) of 0.1. For the fungi *C. neoformans* and *A. fumigatus*, overnight cultures were diluted in 96-well plates to 10^6^ spores per mL as determined by a hemocytometer. The flavanone molecule to be assayed was dissolved in DMSO and then added to 96-well plates in concentration ranging from 0.1 mM up to 2 mM. All flavonoid stocks were 2 mM so serial dilutions (of 1∶2) were used to create the concentration series. The final DMSO concentration was held constant at 5% for all testing. Dilute cell suspenstions in growth media were then added to the wells with a final reaction volume of 100 µL. Each flavonoid compound was also tested in presence of two the efflux pump inhibitors Phe-Arg-ß-naphthylamide (PABN) and 1-(1-Naphthylmethyl)-piperazine (NMP) with concentrations of 25 µg/mL and 100 µg/mL respectively. All assays were done in triplicate with DMSO controls. After assay setup, the OD_600_ of each plate was measured using a BioTek microplate reader and then incubated at 37°C for 24 hours wrapped in paraffin to minimize evaporation with plates being remeasured after incubation. Concentration at which no growth was seen over the incubation period was deemed the Minimum Inhibitory Concentration (MIC) for the compound under investigation. To verify MIC values, dilutions were made to obtain countable colonies from each well after being plated on 10% agar plates made from the respective growth medias. Experimental controls without the flavonoid compound were used throughout testing. The concentration for which the colony-forming unit (CFU) count matched that of the control plates was again deemed the MIC value.

### Cytotoxicity assay

HeLa cell cultures were grown to confluence in supplemented DMEM maintained at 37°C and in a humidified atmosphere (90%) containing 5% CO_2_. Cell were washed once with phosphate-buffered saline (PBS) then briefly incubated with 2 mL of cell dissociation buffer (Invitrogen) to detach adherent cells. Cells were then seeded at 50,000 cells per mL in sterile 96-well flouroscence plates for 24 hours with fresh media. After incubation, 100 µL of the day-old media was replaced by 100 µL of fresh media containing the modified flavanone at various concentrations with the final DMSO concentration adjusted to 0.5% v/v in all conditions. Cell viability measurements were taken after 3 hours and 24 hours of incubation at 37°C using the Cell Titre-Blue Viability Assay kit from Promega as directed by the manufacturer. All compounds were tested with 150 µg/mL in triplicate with the appropriate DMSO only and cell free controls used throughout.

## Results

### Natural flavanones as bacteriostatic agents

To establish a base-line anti-microbial potency of flavanones in general, we first tested the natural flavanones, namely naringenin, pinocembrin and eriodictoyl, for their ability to limit the growth of the gram-negative bacteria *E. coli* (Table 1). Overnight cultures of *E. coli* in rich media failed to significant inhibit growth with natural flavanones using the study's maximum concentrations of 544 µg/mL for naringenin, 512 µg/mL for pinocembrin and 576 ug/mL for eriodictoyl. However, in the presence of the RND efflux pump inhibitors NMP and PABN there was a significant reduction in the growth ability of *E. coli*. To verify that the efflux pump inhibitors were not acting as the growth inhibitory agents, MIC values for NMP and PABN were similarly determined. With the MIC values of 226 µg/mL for NMP and 52 µg/mL for PABN found for the RND inhibitors alone, the respective concentrations (100 µg/mL and 25 µg/mL) used in conjunction with the flavanones for evaluating anti-microbal efficacy can be considered well within a tolerable limit for *E. coli* growth. Similarly, the natural flavanones were tested on the gram-positive bacteria *B. subtilis* both alone and in the presence of NMP (Table 1). The inhibitor PABN was not used against *B. subtilis*. While *B. subtilis* was found to be more susceptible to the natural flavanones alone, they were more effective at limiting growth in the presence of the RND efflux pump inhibitor NMP.

**Table 1:** Natural flavanone MIC for bacteria.

	*E. coli*			*B. subtilis*	
	None	NMP	PABN	None	NMP
Naringenin	>544	544	272	272	136
Eriodictoyl	>576	>576	288	288	144
Pinocembrin	>512	512	256	256	128

MIC values (ug/mL) are reported for natural flavanones against *E. coli* and *B. subtilis* both without and with the efflux pump inhibitors NMP and PABN.

### Flavanone analog synthesis and antibacterial potency

In an effort to improve the bacterial toxicity of flavanones, a small library of non-natural flavanones was created by chemical synthesis, although large-scale synthesis can be accomplished through microbial fermentations in a mutasynthses approach [20]. Synthesis of the non-natural flavanones was completed in four reaction steps, which form protected chalcone intermediates and then follow with de-protection and ring closure. The eight non-natural flavanone analogs constructed include the substitutions 2-flouro- (2F-), 3-hydroxy- (3OH-), 3-flouro- (3F-), 4-chloro- (4Cl-), 4-flouro- (4F-), 4-flouro-3-bromo- (4F-3B-), 4-flouro-3-chloro- (4F-3Cl-) and 4-flouro-3-methoxy- (4F-3OM-). Combinations of 4Cl- and 3F- as well as other substitutions failed to obtain the proper flavanone after synthesis and purification and were not pursued further. Final products verified by HPLC were tested for their antimicrobial potency against a range of microorganisms.

The library was first screened against the bacterial species *E. coli* and *B. subtilis* in 96-well plate growth assays and CFU counting assays (Table 2). Similar to the natural flavanones, the non-natural analogs alone failed to have any potent activity to limit growth of *E. coli*. However in the presence of the RND inhibitor PABN, the potency of the non-natural analogs was significantly increased by up to 7-fold for 4-chloroflavanone. However, *B. subtilis* did show significant sensitivity to the non-natural analogs both with and without the efflux inhibitor NMP (Table 2). Importantly, the MIC values for some compounds approach those of traditional antibiotics such as kanamycin. All results were confirmed by both triplicate cultures in 96-well plates and by CFU counting (data not shown). It is important to note that 3OH-flavanone was found to have a low aqueous solubility (approximately 2 mM), making the potential production and use of this molecule challenging.

**Table 2:** Non-natural flavanone MIC for bacteria.

	*E. coli*			*B. subtilis*	
	None	NMP	PABN	None	NMP
2F-FNN	>548	>548	137	-	-
3F-FNN	>548	>548	137	137	137
3OH-FNN	>546	>546	150	-	-
4F-FNN	>548	>548	137	55	27
4Cl-FNN	>581	581	**70**	20	20
4F-3Cl-FNN	>617	>617	>617	20	20
4F-3B-FNN	>706	706	176	20	**10**
4F-3OM-FNN	>608	304	250	304	80

MIC values (ug/mL) are reported for non-natural flavanones against *E. coli* and *B. subtilis* both without and with the efflux pump inhibitors NMP and PABN.

### Antifungal efficacy non-natural flavanones

To further explore the potency of the flavanone analog library, we tested the ability of these new molecules to inhibit the growth of the non-pathogenic yeast *S. cerevisiae* and the pathogenic fungi *C. neoformans* and *A. fumigatus*. Evaluating the growth inhibition of each species was conducted in a similar manner to the bacterial species but with incubations carried out at 30°C and incubation times extended to account for slower doubling times of fungal species. For *C. neoformans* and *A. fumigatus*, overnight cultures were diluted in 96-well plates to 10^6^ spores per mL. Furthermore, efflux pump inhibitors were not used, as the RND systems are not present in yeast and fungi. The natural flavanones failed to inhibit growth in any of the species at concentrations used in the study (data not shown), with one exception. Eriodictoyl was able to completely inhibit growth of only *S. cerevisiae* with a MIC value of 576 µg/mL. As a positive control, an MIC for yeast with the known antifungal geneticin was identified at 36 µg/mL, which is value slightly lower than the reported 100 µg/mL but may only be due to a difference in strains used.

**Figure 2:**
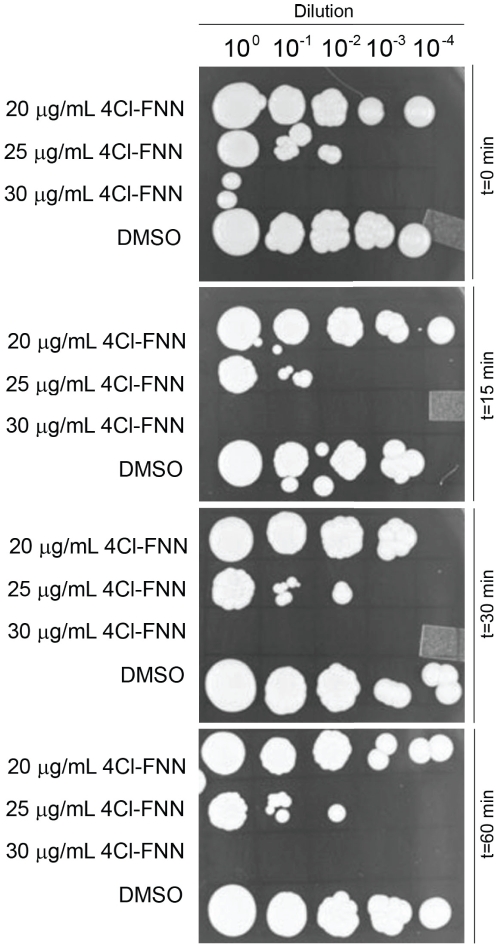
Growth-inhibitory activity of 4-Cl-Flavanone (FNN) against *C. neoformans*. Agar plate spotting in four 10-fold dilutions after cell exposure to 4-chloroflavanone (4Cl-FNN) for 0, 15, 30 and 60 minutes at concentrations of 20, 25 or 30 µg/mL. DMSO was included as a vehicle control.

In contrast, MIC values were found for all of the non-natural flavanones with some having significant antifungal capacity (Table 3). Of the compounds tested, only the 3-hydroxyflavanone failed to provide significant antifungal ability on any of the strains tested. The yeast *S. cerevisiae* was found to be highly sensitive to 4-chloroflavanone (30 µg/mL) and moderately sensitive to two others (3-fluoro- and 4-fluoro-) with a MIC value of 55 µg/mL. The most resistant of the three fungal species was *A. fumigatus* and showed only limited growth inhibition with MIC values all greater than 130 µg/mL. One the other hand, three of the four non-natural flavanones tested demonstrated MIC values of approximately 30 µg/mL for the pathogenic *C. neoformans*. Figure 2 shows serial dilution on agar growth plates of *C. neoformans* under different exposure times to 4-chloroflavanone and indicates that the effect is not inhibitory but fungacidal.

**Table 3:** Non-natural flavanone MIC for fungi and yeast.

	*C. neoformans*	*A. fumigatus*	*S. cerevisiae*
Geneticin	-	-	36
Eriodictoyl	-	-	576
3F-FNN	55	274	55
3OH-FNN	>540	>546	>546
4F-FNN	**27**	**137**	55
4Cl-FNN	30	145	**30**
4F-3Cl-FNN	31	617	>617
4F-3B-FNN	71	>706	35
4F-3OM-FNN	608	304	152

MIC values (ug/mL) are reported for non-natural flavanones against the yeast *S. cerevisiae* and fungi *C. neoformans* and *A. fumigatus*.

### Effect of drugs on cell viability

**Figure 3:**
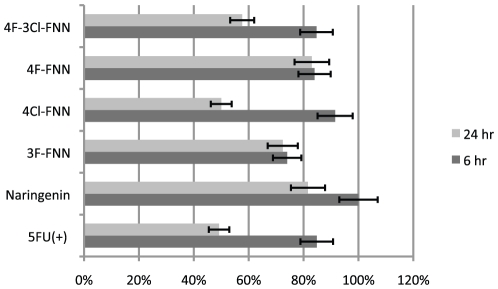
HeLa cell toxicity to non-natural flavanones. Relative cell viability for HeLa cells after 3 hour and 24 hour incubations in the presence of the natural flavanone naringenin and non-naturals 3-fluoro- (3F-), 4-chloro- (4Cl-), 4-fluoro- (4F-), and 4-fluoro-3-chloro- (4Cl-3Cl-) flavanones (FNN). 5-fluorouracil (5FU) was used as a positive control.

One of the challenges of antifungal therapies is that the close phylogenetic reslationship between fungi and mammals often results in cross-kingdom toxicity. Novel antifungal agents thus must ideally exhibit potent antifungal activity with minimal effect on human cell viability. To address the question of cytotoxicity, cell viability assays of the chemically synthesized drugs were performed with the natural flavanone naringenin used as a benchmark for cell toxicity. As a positive control, 5-fluorouracil (5FU) was used and is widely applied chemotherapeutic agent in gastrointestinal carcinoma [29]. Naringenin deemed to be relatively non-toxic with cell viability at 81.6% after 24 hours (Figure 3) using 150 µg/mL, however the 50% Lethal Dose (LD_50_) value for 5-FU is 170 µg/mL at 24 hours. Importantly, while the non-natural compounds were shown to be potent anti-microbial agents, the cyotitoxicity was relatively low. The non-natural analogs with fluorine substitutions were less toxic having cell viabilities greater than 70% after 24 hours while those with chlorine substitutions were relatively more cytotoxic.

## Discussion

Antimicrobial resistance continues to evolve presenting serious challenges to current medical practices to treat community-acquired infections. Often the most talked about infections are bacterial in nature from both gram-positive and gram-negative species [30]. Hoever, fungal species also pose a serious burden on our healthcare system. For example, *Cryptococcus neoformans* is a major human fungal pathogen that particularly affects those with compromised immune systems such as AIDS patients or organ transplant recipients [31]. While only 7–15% of U.S. AIDS patients develop cryptococcal infections, some areas of Africa, such as Zimbabwe, have infection rates as high as 88% [32]. This makes developing new antifungal agents a critical endeavor as cryptococcal species are rapidly developing resistance to current compounds.

This work aimed to identify the potency of flavanones, both naturally occurring and chemically synthesized non-natural analogs, to limit or stop the growth selected microorganisms relevant to the medical community. Chemical modifications where made to the B-ring of the flavanone, a position that has been implicated in its biological activity [33], [34]. Since halogens are not common functional groups in nature, we looked to incorporate these molecules into a variety of B-ring positions. While a small variety of compounds was formed by chemical synthesis here, these molecules have been routine synthesize from microbial fermentations. Such a mutasynthesis route may be able to generate a much more diverse library of compounds, with protein engineering used to expand substrate specificity, and be able to yield significantly larger quantities for broader spectrum testing.

The small collection of flavonoid derivatives tested in this study was found to have up to a 15-fold decrease in MIC value against bacteria when compared to natural flavanones and in the presence of efflux pump inhibitors. The reduced MIC value for all compounds in the presence of RND pump inhibitors illustrates that these molecules are similarly transported across the cell membrane as other antibacterial agents. Lower MIC values, as high as a 10-fold reduction, were also seen for the non-natural analogs against fungi. Furthermore, most of the compounds displayed broad antimicrobial activity against all species tested with one (4-chloroflavanone) showing significant antibacterial activity against both bacteria and fungi. Finally, by verifying that these non-natural flavanones displayed little to no toxicity to mammalian cells, it shows a greater potential to used them as drug candidates for further functional development and clinical testing.

In summary, this data suggest two opportunities for the development of new antimicrobial therapeutics. First, combining antibacterial compounds with efflux pump inhibitors is an efficient way to increase the potency of the therapeutic agent. And secondly, that introducing a variety of novel substitutions to flavonoids, and other natural products in general, can create new compounds that possess different discrete effects between fungi and humans.
